# Surgical Technique and Perioperative Outcomes of the “Sapienza” Urology Residency Program’s Trocar Placement Configuration During Robotic-Assisted Radical Prostatectomy (RARP): A Retrospective, Single-Centre Observational Study Comparing Experienced Attendings vs. Post-Graduate Year I–III Residents as Bedside Assistants

**DOI:** 10.3390/cancers17010020

**Published:** 2024-12-25

**Authors:** Valerio Santarelli, Dalila Carino, Roberta Corvino, Stefano Salciccia, Ettore De Berardinis, Wojciech Krajewski, Łukasz Nowak, Jan Łaszkiewicz, Tomasz Szydełko, Rajesh Nair, Muhammad Shamim Khan, Ramesh Thurairaja, Mohamed Gad, Benjamin I. Chung, Alessandro Sciarra, Francesco Del Giudice

**Affiliations:** 1 Department of Maternal Infant and Urologic Sciences, “Sapienza” University of Rome, 00185 Rome, Italy; dalila.carino@uniroma1.it (D.C.); roberta.corvino@uniroma1.it (R.C.); stefano.salciccia@uniroma1.it (S.S.); ettore.deberardinis@uniroma1.it (E.D.B.); alessandro.sciarra@uniroma1.it (A.S.); 2 Department of Minimally Invasive and Robotic Urology, University Center of Excellence in Urology, Wroclaw Medical University, 50-556 Wroclaw, Poland; wojciech.krajewski@umw.edu.pl (W.K.); tomasz.szydelko@umw.edu.pl (T.S.); 3 Guy’s and St. Thomas’ NHS Foundation Trust, Guys Hospital, London SE1 9RT, UK; rajesh.nair1@gstt.nhs.uk (R.N.); shamim.khan@gstt.nhs.uk (M.S.K.); ramesh.thurairaja@gstt.nhs.uk (R.T.); mohamed.gad@gstt.nhs.uk (M.G.); 4 Department of Urology, Stanford University School of Medicine, Stanford, CA 94305, USA; bichung@stanford.edu

**Keywords:** urology, robotic-assisted, RARP, laparoscopy, training, residency

## Abstract

**Background/Objectives**: Robot-assisted radical prostatectomy (RARP) for the treatment of prostate cancer (PCa) has been standardized over the last 20 years. At our institution, only n = 3 rob arms are used for RARP. In addition, n = 2, 12 mm lap trocars are placed for the bedside assistant symmetrically at the midclavicular lines, which allows for direct pelvic triangulation and greater involvement of the assisting surgeon. The aim of our study was to compare surgical and perioperative outcomes of RARP performed using our alternative trocar placement with no fourth robotic arm in the subgroups of experienced attending surgeons and post-graduate residents as bedside assistants. Residents’ satisfaction was also explored. **Methods**: RARPs performed within the urology residency program between 2019 and 2024 were retrospectively analyzed. Only rob procedures performed using our 3+2 trocars configuration were included. Intra- and postoperative outcomes, as well as long-term functional outcomes including continence recovery and potency, were assessed, stratified by the level of expertise of the bedside assistant, i.e., an experienced attending or post-graduate Year I–III resident. Satisfaction of residents assigned to the two groups during their robotic rotation was evaluated considering three domains with a score from 1 to 10: insight into surgical procedure, confidence level, and gratification level. **Results**: Out of n = 281 RARP procedures, the bedside assistant was an attending in 104 cases and a resident in 177. Operative time was found to be slightly longer in cases where the second operator was a resident (attendings vs. residents: 134 ± 40 vs. 152 ± 24; *p* < 0.001). Postoperative hospitalization time was longer in patients in the resident group (attendings vs. residents: 3.9 ± 1.6 vs. 4.3 ± 1 days; *p* = 0.025). However, cases where the second operator was a resident had a lower rate of positive surgical margins, with rates of 19.7% in the resident and 43.3% in the attending surgeon cohorts (OR = 0.32; 95% CI 0.18–0.55). This difference remained significant in multivariate analysis. There was no significant difference in postoperative blood transfusion rates (attendings vs. residents: 1.9% vs. 1.2%; *p* = 0.6). Similarly, long-term functional outcomes in terms of erectile dysfunction and urinary incontinence rates mostly overlapped between groups. The mean score in all three domains evaluating residents’ satisfaction was significantly higher when residents actively participated in the surgical procedure as bedside assistants (*p* = 0.02, *p* = 0.004, and *p* < 0.001, respectively, for insights into surgical procedure, confidence level, and gratification level). **Conclusions**: These findings provide insight into how an alternative port positioning during RARP could improve the involvement of the bedside assistant, particularly residents, without compromising perioperative outcomes or surgical safety.

## Simple Summary

Robot-assisted radical prostatectomy (RARP) has been standardized for prostate cancer (PCa) over the last 20 years. Using the Sapienza residency program 3+2 trocar configuration with a second laparoscopic port replacing the fourth robotic arm, the bedside assistant is more involved during the surgical procedure and requires higher laparoscopic skills. In this study, we examine perioperative, functional, and oncological results of RARP using the Sapienza 3+2 trocar configuration in cases where the bedside assistant was either an attending surgeon or a urology resident. We identified that the level of expertise of the bedside assistant did not influence oncological and functional results. With this premise, the Sapienza 3+2 trocar configuration could be employed in residency programs to allow laparoscopic training of surgical residents without compromising the current request for robotic-assisted surgeries.

## 1. Introduction

Prostate cancer (PCa) is the second most commonly diagnosed malignancy in male patients, with 1.4 million new diagnoses worldwide in 2020 [1]. Thanks to widespread screening and early detection rates, PCa mortality has decreased in most Western nations, but its high prevalence still makes it the third cancer-related cause of death for European men [2,3]. Androgen-deprivation therapy (ADT), eventually associated with chemotherapy or androgen receptor signaling inhibitors (ARSI), is the standard treatment option for metastatic PCa [4,5]. In the case of localized and locally advanced PCa, radiation therapy and radical prostatectomy (RP) with or without extended pelvic lymph node dissection (ePLND) are the two more relevant therapeutic options [6]. RP can be performed with an open, laparoscopic, or robotic-assisted approach. Compared to open RP (ORP), laparoscopic RP (LRP) and robotic-assisted RP (RARP) have demonstrated fewer complication rates and better oncological and functional outcomes [7,8]. With the increasing availability of surgical robots in high-volume centers, RARP has become the most frequently performed approach for the treatment of non-metastatic PCa. While the advantages of a laparoscopic approach, either standard or robotic-assisted, over an open approach are well accepted, the European Urological Association (EAU) recommends informing candidates for RP that neither laparoscopic nor robotic approach has shown a clear superiority in terms of functional and oncological outcomes [9]. Despite that, the better precision and vision of a robotic approach, together with a shorter learning curve, makes it the favored approach for both expert and young surgeons [10,11]. While an open approach is still recommended in some cases where a pneumoperitoneum is contraindicated, laparoscopy and robotic approaches share virtually the same contraindications [12].

In this scenario, young surgeons and surgical residents are more likely to be exposed to robotic surgery without proper laparoscopic training. However, there are many reasons why laparoscopic training should not be abandoned. Firstly, laparoscopic surgery is still very common worldwide, and data suggest its popularity is still on an ascending phase in many countries of the European Union (EU) [13]. Secondly, while the diffusion of surgical robots is increasing, there are still many centers that practice only laparoscopic surgery, and it is unlikely that robotic technology will be able to cover them all. Thirdly, robotic-assisted operations still have higher costs compared to laparoscopic operations [14]. Fourthly, the management of some complications that might occur during a robotic procedure still requires high laparoscopic skills.

The four-arm approach is the classical trocar placement method for RARP. The camera port is placed about 1.5 cm above the umbilical scar at the midline. One robotic port is placed on the left side, five fingerbreadths from midline, and two robotic ports are placed on the right side, four fingerbreadths (1 fingerbreadth ~1.9 cm) apart from each other. A 12 mm assistant port is normally placed halfway between the camera port and the left robotic port. Another 5 mm or 12 mm is normally maintained laterally to the left robotic trocar. In most centers, the majority of the instruments (e.g., bipolar scissors, monopolar forceps, clip appliers, vessel sealers, and needle drivers) are robotic. The table-side assistant (usually a trained nurse rather than an attending surgeon or a surgical resident) is responsible for passing sutures, changing robotic instruments, and suction–irrigation and only operates the suction set and a Johan grasper.

In this article, we present a new trocar configuration for a transperitoneal RARP approach using the DaVinci X or Xi system. With the Sapienza residency school trocar configuration, two 12 mm laparoscopic arms are placed at the level of the midclavicular line, allowing ideal triangulation of the pelvis and sparing the fourth robotic arm. The only robotic instruments used are the bipolar scissors, monopolar forceps, and needle driver. The bedside operator plays a crucial role in aspiration, traction, dissection with the laparoscopic vessel sealer, dynamic suspension of the prostate during posterior dissection (normally carried out statically by the fourth arm with the ProGrasp), bleeding, and neurovascular bundle control with the laparoscopic clip applier. The aim of this study is to retrospectively analyze complication rates and oncological and functional outcomes of RARP with or without ePLND for procedures performed at our institution with the Sapienza residency trocar configuration, stratified by the level of expertise of the bedside assistant (an experienced attending or a post-graduate Year I–III resident). In addition, we compare the satisfaction of residents assigned to the two surgical groups.

## 2. Materials and Methods

### 2.1. Population

Patients with a histologically confirmed diagnosis of non-metastatic PCa submitted to RARP with the Sapienza 3+2 trocar configuration were included. All patients were discussed by a multi-disciplinary team (MDT) prior to being offered the possible therapeutic options according to EAU guidelines [9]. Active surveillance (AS), surgery, and RT were the three options presented to low-risk cases, while intermediate or high-risk patients were offered either RT or surgery. The possible advantages, limitations, and side effects of the presented options were explained. All included patients chose RARP and signed informed consent for the procedure. Inclusion criteria were the absence of distant metastases at clinical staging, histological diagnosis of prostate cancer, estimated life expectancy of 10 years or longer, and RARP as the chosen primary therapeutic option. Exclusion criteria were previous chemotherapies, pelvic radiation therapies, androgen deprivation therapies, or treatment with any medication that could impact prostate tumor growth. Prior to surgery, all patients underwent prostate-specific antigen (PSA) level determination and prostate biopsy. In cases that also underwent multiparametric magnetic resonance imaging of the prostate (mpMRI) with PI-RADS score determination prior to surgery (the majority), targeted sampling was performed in addition to the standard random biopsy scheme. PSA level, clinical stage according to digital rectal examination (DRE) or mpMRI, and Gleason score (GS) were used to stratify patients according to the EAU D’Amico risk groups [15]. In all intermediate- and high-risk patients, systemic staging was performed. Validated nomograms predicting the risk for positive lymph node involvement were used [16].

### 2.2. Groups Description

At present, at the Sapienza University urology residency school, urology residents are assigned to either one of two surgical groups during their robotic rotation in the first three years of residency. Residents assigned to the first group (the attending group), while still attending the operating room (OR), are less likely to participate in surgical operations. When scrubbed up during RARP, they may help with trocar positioning and robot docking but do not actively engage in the surgical procedure. In this group, the bedside assistant is always an attending surgeon. In the second group (the resident’s group), attending surgeons supervise surgical residents during access, trocar positioning, and bedside assistance and rarely intervene unless deemed necessary.

### 2.3. Surgical Procedure

**Figure 1:**
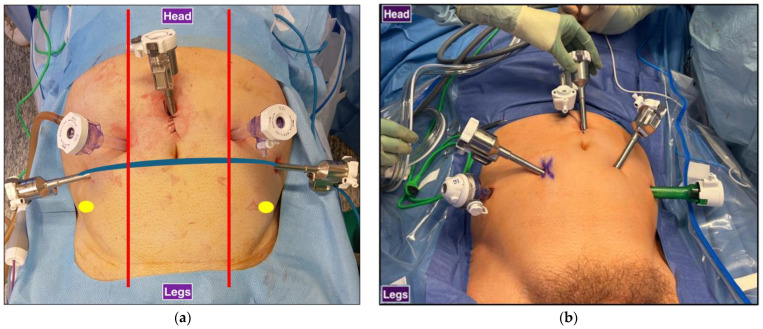
Trocar configurations for RARP: (**a**) Sapienza residency school 3+2 trocar configuration. Laparoscopic trocars are symmetrical, and the assistant is at the head of the patient. Red: midclavicular line, blue: transumbilical plane, yellow: anterior superior iliac spine. (**b**) Standard 4+2 trocar configuration. Laparoscopic trocars are not symmetrical, and the assistant is standing at the side of the surgical bed.

**Figure 2:**
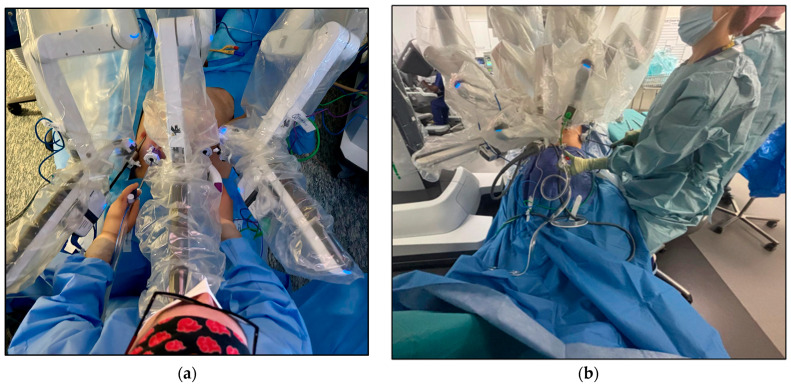
Bedside assistant position during RARP (**a**) With the Sapienza residency school 3+2 trocar configuration, the bedside assistant is positioned at the head of the patient for better triangulation and access to the patient’s pelvis. (**b**) With the standard 4+2 trocar configuration, the bedside assistant stands laterally to the surgical bed and is less involved in the surgical procedure.

**Figure 3:**
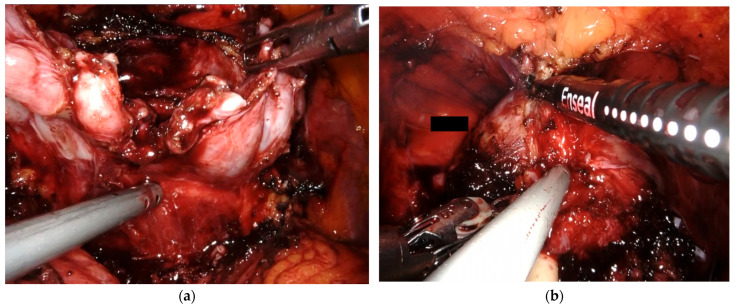
Intraoperative photos (**a**) With the Sapienza residency school 3+2 trocar configuration, the bedside assistant has direct access to the patient’s pelvis, which allows for direct involvement throughout the procedure. (**a**) The bedside assistant is using a Johan grasper in the right hand for backward and upward traction while aiding posterior dissection with the aspirator in the left hand. (**b**) The bedside assistant employs the vessel sealer for dorsal vein complex (DVC) management.

All included patients underwent RARP with the 3+2 Sapienza trocar configuration with either an attending surgeon or a post-graduate year (PGY) I–III urology resident as the bedside assistant. The Sapienza residency school trocar configuration for RARP is shown in Figure 1 and Figure 2. The camera port is placed 2–3 cm above the umbilical scar. Two 8 mm robotic ports are placed laterally at the level of the umbilical scar. One laparoscopic 12 mm port and the Airseal 12 mm port are placed symmetrically, slightly lower than the level of the camera port, four fingerbreadths from both the robotic trocar and the camera port and at the level of the midclavicular line. The fourth robotic arm is not employed. The bedside assistant is positioned at the head of the patient, with shoulders and instruments in line with the robotic arms and facing the patients’ pelvis for better triangulation (Figure 3). With this trocar configuration, while the main operator is still the surgeon operating the DaVinci console, the bedside operator is a medical doctor (MD), either a post-graduate resident or an attending surgeon. The only robotic instruments used are the bipolar scissors, monopolar forceps, and needle driver. Instead of their robotic counterparts, a laparoscopic vessel sealer and a clip applier are used.

All procedures were performed in a single institution using the same intraperitoneal standard technique for RP. Each group had the same primary surgeon, and both primary surgeons had a high level of expertise for robotic procedures (>5 years). An ePLND was performed in all high-risk cases and all intermediate or low-risk cases with an estimated risk of lymph node involvement ≥ 5%. A nerve-sparing (NS) technique (intrafascial, unilateral, or bilateral) was chosen in suitable cases after discussion with the patient based on the possible risks and the likelihood of maintaining potency. Operative time and any intra- or perioperative complications, such as anastomotic leakage, lymphocele, rectal injury, or transfusion, were reported. Catheterization time and hospitalization time were also recorded.

### 2.4. Oncological and Functional Outcomes

pT stage according to the International Society of Urological Pathology (ISUP) grading, perineural invasion (PNI), and cribriform differentiation at final pathology were reported. Follow-up visits were scheduled every 3 months for the first two years and then every 6 months. In case of recurrent disease, time to biochemical (total PSA ≥ 0.2 confirmed by blood test) or radiological (confirmed by mpMRI or PET CT scan) local or distant recurrence was reported. Postoperative functional complications, such as urethral stricture, urinary incontinence (UI), and erectile dysfunction (ED), were reported during a 12-month follow-up. UI was defined as persistent urinary leakage ≥ 5 g in a 24 h pad test. An International Index of Erectile Function-5 (IIEF-5) score between 5 and 10 was used to define clinically significant ED.

### 2.5. Residents’ Satisfaction

Residents assigned to the two groups were asked to retrospectively assess their level of satisfaction during their robotic rotation in the first three years of residency. Three domains were evaluated—insight into surgical procedure, confidence level, and gratification level—with a score from 1 to 10, with 1 being the least satisfied and 10 being the most satisfied.

### 2.6. Statistical Analysis

SPSS Statistics (IBM Corp: Armonk, NY, USA) version 27.0 program was used for statistical analysis. Descriptive statistics were expressed as the number of cases, mean ± SD, median, and range. Qualitative data were compared using a Fisher’s exact test and a chi-square test. For quantitative data comparison and pairwise intergroup comparisons of variables, a Mann–Whitney test or an ANOVA one-way test was used. Univariate and multivariate Cox proportional analyses for clinical and pathological parameters were performed. Statistical significance was evaluated at *p* < 0.05. Kaplan–Meier analysis was carried out to compare the rate of development of biochemical recurrence.

## 3. Results

### 3.1. Description of the Population

#### 3.1.1. Patient Characteristics

Baseline characteristics of the whole population are shown in Table 1. Of the 281 cases, 104 belonged to the attending group and 177 to the resident group. The mean age of the population was 64.54 ± 6.35 with a range of 47 to 75 years, and the mean preoperative BMI and PSA were 26.38 ± 3.55 and 8.76 ± 5.94 (range 1.69–50), respectively.

Intermediate- and high-risk PCa, according to the D’Amico risk group classification, were found in 44.1% and 27% of cases, respectively. A total of 200 (78.7%) patients who underwent a preoperative mpMRI had lesions classified as PIRADS 4 or 5. Of the whole population, 227 (80.7%) patients had a preoperative cT stage of cT2, while 39 (13.9%) and 4 (1.4%) patients were cT3a and cT3b, respectively. An NS approach was performed in 48.7% (n = 137) of cases, while an ePLND was performed in 36% (n = 101) of the total cohort. Mean surgical operative time was 145.34 ± 32.19 min (range 65–320), mean hospitalization time was 4.15 ± 1.28 days (range 2–13 days), and mean time of catheterization was 10.23 ± 7.12 days (range 6–122 days). At final pathology, extracapsular disease (pT3) was found in 43.4% of cases (pT3a 34.5% and pT3b 8.9%), 28.6% (n = 79) of patients had positive surgical margins, and 23% (n = 24) of patients submitted to an ePLND had 1 or more positive lymph nodes. Regarding postoperative complications, the rates of urethral stricture, urinary incontinence (UI), and erectile dysfunction (ED) were 1.4%, 17.8%, and 38.8%, respectively. Moreover, 28 (9.8%) patients developed biochemical progression, with a mean time to biochemical recurrence (BCR) of 6.65 ± 7.2 months (range 2–36 months).

#### 3.1.2. Differences in Preoperative Parameters

Clinical parameters such as age and BMI were not significantly (*p* > 0.050) different between the attending and resident groups. Patients in the attending group had a significantly higher mean preoperative PSA level than those in the resident group (9.76 ± 7.28 vs. 8.18 ± 4.92, *p* = 0.032). The distribution of PIRADS 1–5 cases at mpMRI and ISUP 1–5 cases at preoperative biopsy did not vary significantly between the two groups. Clinical stages T3a and T3b were found in 11.9% (n = 21) and 1.7% (n = 3) of patients in the resident group, respectively, and in 17.3% (n = 18) and 1% (n = 1) of patients in the attending group (Table 1). High-risk PCa cases were more frequent in the attending group than in the resident group (34.6% vs. 22.6%), but the difference did not reach significance (*p* = 0.063).

#### 3.1.3. Differences in Surgical Procedure

Cases in the resident group had a significantly longer mean operative time than those in the attending group (152.33 ± 24.1 vs. 134.38 ± 39.55, *p* < 0.001). A NS approach was more frequently performed in the attending group than in the resident group (*p* = 0.002). When the surgeon opted for an NS approach, a bilateral NS procedure was the most frequent choice in the resident group (bilateral n = 42, unilateral n = 31), while a unilateral NS approach was more often preferred in the attending group (unilateral n = 33, bilateral n = 31). There was no significant difference in the rate of ePLND performed between the two groups (*p* = 0.74). The mean postoperative hospitalization time was shorter in the attending group (3.93 ± 1.63 days vs. 4.3 ± 1.4 days, *p* = 0.025), while there was no significant difference in terms of catheterization time (*p* = 0.83).

#### 3.1.4. Differences in Oncological and Functional Outcomes

At final pathology, ISUP grading and PNI rate did not differ significantly between the two groups, while cribriform differentiation was more frequent in the attending group (*p* < 0.001). Pathological T stage and N stage were similar in the two groups (*p* = 0.374 and *p* = 0.41, respectively). On the contrary, there was a significant difference in terms of SM+ rates. A positive surgical margin was found in 43.3% (n = 45) of patients in the attending group and in 19.7% (n = 34) of cases in the resident group (*p* < 0.001). Patients in the attending group had a higher rate of BCR with a shorter BCR time, but the difference did not reach significance (*p* = 0.25 and *p* = 0.48). Regarding functional outcomes, there was no significant difference in terms of urinary stricture, UI, or ED rates between the two groups.

### 3.2. Perioperative Outcomes Stratified by the Level of Expertise of the Bedside Assistant

Table 2 shows a logistic regression analysis that was used to assess how the experience of the bedside assistant impacted adverse pathological, oncological, and functional outcomes in our population of non-metastatic PCa patients submitted to RARP. For the variables included in the analysis, with the attending group as the reference category, having a resident as the bedside assistant did not significantly increase the risk of BCR, urinary stricture, ED, and UI. Contradictorily, patients in the resident group had a lower risk of a positive surgical margin at pathology (OR = 0.32, 95% CI 0.18–0.55, *p* < 0.001). On multivariate analysis, after adjusting for preoperative PSA, risk class, biopsy multifocality, and cT stage, SM positivity remained the only variable independently and significantly associated with the bedside assistant level of expertise.

### 3.3. Survival Analysis Stratified by the Level of Expertise of the Bedside Assistant

**Figure 4:**
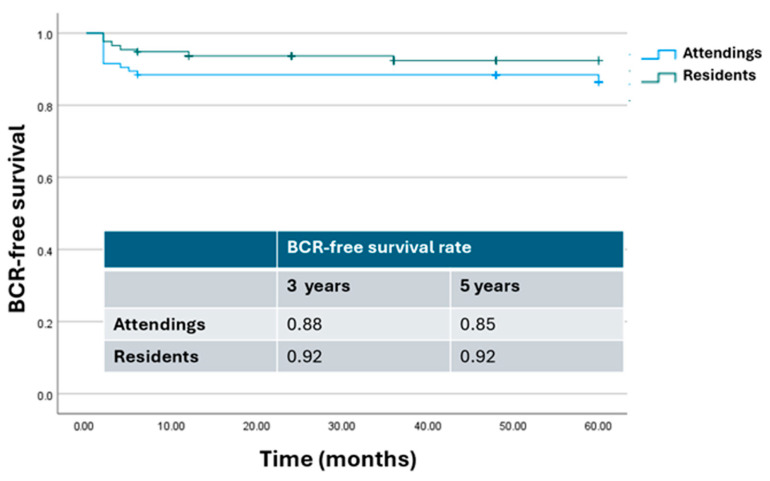
Kaplan–Meier analysis. Estimated rates of biochemical-free survival (BFS) according to the level of expertise of the bedside assistant. Attendings = attending surgeon as bedside assistant; residents = resident as bedside assistant.

The Kaplan–Meier curve describing cumulative BCR-free survival rates according to the bedside assistant level of expertise at RARP is shown in Figure 4. As can be seen, there was no significant difference in terms of BCR-free survival rate at 3 and 5 years after surgery (*p* = 0.4 and *p* = 0.25, respectively).

### 3.4. Differences in Residents’ Satisfaction

Residents’ satisfaction was evaluated with a questionnaire assessing three different domains with a score from 1 to 10: insight into surgical procedure, confidence level, and gratification level. A total of 68 residents participated in the analysis, 32 in the attending group and 36 in the resident group. Results are shown in Table 3. The mean score was higher in the residents’ group for all three domains (7.8 ± 2.2 vs. 6.3 ± 2.8 for insight into surgical procedure, *p* = 0.02; 6.9 ± 1.6 vs. 5.1 ± 2.9 for confidence level, *p* = 0.004; 8.3 ± 1.6 vs. 6.1 ± 1.8 for gratification level; *p* < 0.001).

## 4. Discussion

In recent years, the surgical field has undergone a remarkable transformation thanks to the rise of minimally invasive techniques. Since its development, laparoscopic surgery has become the technique of choice for virtually every kind of abdominal surgery. The advantages of laparoscopic surgery include better aesthetic results, lower postoperative pain, lesser estimated blood loss (EBL), and earlier hospital discharge [17,18,19,20,21]. However, traditional laparoscopy shows several limitations, such as the transmission of physiologic tremors, limited degrees of freedom, two-dimensional vision, the fulcrum effect, and so on [17]. In particular, when it comes to LRP, the limitations of traditional laparoscopy become evident during the vesicourethral anastomosis. For this reason, LRP is considered a complex procedure with a steep learning curve. The introduction of RARP, characterized by enhanced ergonomics, three-dimensional magnified vision, and improved maneuverability within the constricted pelvic cavity, has facilitated the swift global adoption of robotic techniques [22,23,24,25]. The use of a robotic approach also helps flatten the steep learning curve of the laparoscopic version. However, the prohibitively high costs of RARP compared to LRP and the lack of clear evidence supporting one technique over the other make LRP still very popular in Europe and Asia [26,27]. A systematic review of two RCTs comparing LRP with RARP revealed no significant differences in EBL, blood transfusion rates, positive surgical margin (SM) rates, or mean length of stay (LOS) between the two methods [28]. BCR-free survival was also similar between the two approaches, while RARP was found to have significantly higher return to erectile function and continence rates. According to current international guidelines, no specific surgical approach can be deemed superior [9]. Instead of prioritizing a particular surgical technique, the experience of the surgeon and hospital volume may play a more significant role in achieving optimal functional and oncological outcomes. In this context, with the continued popularity of traditional laparoscopy throughout the European Union (EU) [13], laparoscopic training should not be regarded as an obsolete or discretionary component of urology resident education. With the advancement of robotic-assisted procedures and the relegation of laparoscopic techniques, especially in large university hospitals with normally higher funds to afford surgical robots, urology residents tend to seek and find laparoscopy training in the form of two-day or weekend courses [29]. Obviously, without subsequent adequate and independent training and mentorship during the residency training program, short courses are not sufficient alone for ideal and safe incorporation of laparoscopy into a surgeon’s repertoire.

In this article, we presented a novel trocar configuration for transperitoneal RARP using the DaVinci X or Xi system. With the Sapienza residency training program’s 3+2 trocar configuration, the bedside assistant is always an MD, either a resident or an attending surgeon, and has a crucial role throughout the surgical procedure. Without utilizing the fourth robotic arm and minimizing the use of robotic clip appliers and sealers, the bedside operator remains actively involved throughout all surgical steps, closely resembling the experience of performing a traditional laparoscopic procedure. This engagement not only enhances the operator’s skill set but also fosters a collaborative environment in the operating room, allowing for real-time decision-making and picking up hands-on technical adjustments. After adequate training, the bedside assistant will not only take on the role of the fourth robotic arm but also evolve it from a static–passive traction to a dynamic, ever-adjusting assistance. With the bedside assistant working in sync with the primary surgeon and avoiding the surgical pauses required to allow adjustment of the position and traction of the fourth arm, the surgical procedure becomes more fluid, and the primary surgeon can stay focused longer throughout the operation. In addition, we evaluated the differences in terms of surgical, oncological, and functional outcomes of having a PGY I–III urology resident or an attending surgeon as a bedside assistant using the Sapienza 3+2 trocar configuration. We found no differences in terms of complication rates, oncological outcomes expressed as BCR-free survival rates, or functional outcomes, specifically ED and UI rates. We found that cases in the resident group had longer operating times and were more likely to have negative SM. This difference can be attributed to the different main operator. The operator in the attending group tended to go faster but was less careful during surgical dissection. As a matter of fact, previous research has demonstrated an association between higher positive SM rates and shorter operating times [30]. In addition, the higher rate of NS approach in the attending group could also explain the difference in SM positivity. Lastly, we evaluated surgical residents’ satisfaction in the two groups. Satisfaction was compared using three domains (insight into surgical procedure, confidence level, and gratification level) with a score from 1 to 10, with 1 being the least satisfied and 10 being the most satisfied. Residents in the resident group demonstrated higher mean scores in all three domains. The more direct and active participation of residents in the latter group likely helped enhance their understanding of the surgical procedure, building confidence and providing a stronger sense of gratification. On the contrary, the lower contribution to the surgical process of the residents in the attending group negatively impacted not only their gratification and confidence level but also their comprehension of the operation.

We acknowledge several limitations to our study. This is a retrospective analysis, and the population characteristics were not uniform across the two groups. Additionally, as the main operator differed between the groups, it is difficult to evaluate the impact of this variability on the results. While we compared the effectiveness of the Sapienza 3+2 trocar configuration for RARP with either an attending or a resident as the bedside assistant, this single-center study did not allow us to compare the Sapienza 3+2 configuration with the standard 4+1/2 configuration. While we were able to empirically assess residents’ satisfaction in the two groups, we could not demonstrate that assistance with the Sapienza 3+2 trocar configuration during RARP improved laparoscopic skills compared to the standard 4+1/2 configuration. Moreover, residents’ satisfaction was not evaluated with a validated questionnaire. Additional research is needed. First, a prospective, ideally randomized, multi-center study should compare the standard 4+1/2 trocar configuration with the Sapienza 3+2 configuration for RARP. Second, a prospective study should objectively assess both laparoscopic skills and satisfaction levels in residents who have assisted in multiple procedures using either the Sapienza 3+2 or the standard 4+1/2 configuration.

## 5. Conclusions

In this article, we presented a novel trocar configuration for the execution of an intraperitoneal RARP. Using the Sapienza residency program 3+2 trocar configuration with a second laparoscopic port instead of the fourth robotic arm, the bedside assistant is much more involved during the surgical procedure and requires higher laparoscopic skills. In the present study, having a resident as the bedside assistant was not inferior to having an attending surgeon in terms of oncological and functional results. With these premises and after further validation, the Sapienza 3+2 trocar configuration could be employed in residency programs to allow laparoscopic training of surgical residents without compromising the results of the operation.

## Figures and Tables

**Table 1:** Distribution of preoperative and postoperative parameters on the basis of bedside assistant expertise level.

	All Cases	Attending Group	Resident Group	*p* Value
Number cases	281	104	177	
Age (years)	64.54 ± 6.35; 66 (47–75)	63.8 ± 6.63; 65 (47–75)	65 ± 6.16; 66 (47–75)	0.127
BMI	26.38 ± 3.55; 26 (18.51–40.46)	26.67 ± 3.77; 26 (19–39)	26.21 ± 3.41; 26 (18.51–40.46)	0.297
Preoperative total PSA (ng/mL)	8.76 ± 5.94; 6.9 (1.69–50)	9.76 ± 7.28; 7.6 (1.7–50)	8.18 ± 4.92; 6.9 (1.69–27)	0.032
mpMRI PIRADS score				0.097
PIRADS 1–2	2 (0.8%)	2 (2.6%)	0 (0%)	
PIRADS 3	52 (20.5%)	16 (20.8%)	36 (20.3%)	
PIRADS 4–5	200 (78.7%)	59 (76.6%)	141 (79.7%)	
Clinical T staging				0.01
T1	11 (3.9%)	10 (9.6%)	1 (0.6%)	
T2a	14 (5%)	3 (2.8%)	11 (6.2%)	
T2b	123 (43.7%)	32 (30.8%)	91 (51.4%)	
T2c	90 (32%)	40 (38.5%)	50 (28.2%)	
T3a	39 (13.9%)	18 (17.3%)	21 (11.9%)	
T3b	4 (1.4%)	1 (1%)	3 (1.7%)	
ISUP grading at biopsy				0.093
1	78 (27.8%)	24 (23.1%)	54 (30.5%)	
2	111 (39.5%)	45 (43.3%)	66 (37.3%)	
3	54 (19.2%)	20 (19.2%)	34 (19.2%)	
4	24 (8.5%)	6 (5.8%)	18 (10.7%)	
5	14 (5.0%)	9 (8.7%)	5 (2.8%)	
Risk class (D’Amico)				0.063
Low risk	81 (28.8%)	29 (27.9%)	52 (29.4%)	
Intermediate risk	124 (44.1%)	39 (37.5%)	85 (48%)	
High risk	76 (27%)	36 (34.6%)	40 (22.6%)	
Operative time (min)	145.34 ± 32.19; 140 (65–320)	134.38 ± 39.55; 135 (65–320)	152.33 ± 24.1; 145 (90–300)	<0.001
Nerve sparing technique at surgery				0.002
No	144 (51.2%)	40 (38.5%)	104 (58.7%)	
Unilateral	64 (22.7%)	33 (31.7%)	31 (17.5%)	
Bilateral	73 (26%)	31 (29.8%)	42 (23.7%)	
Extended lymph node dissection				0.74
No	180 (64%)	68 (65.4%)	112 (63.3%)	
Yes	101 (36%)	36 (34.6%)	65 (36.7%)	
Pathological stage (T)				0.374
pT2	160 (56.9%)	63 (60.6%)	97 (54.8%)	
pT3a	97 (34.5%)	31 (29.8%)	65 (36.7%)	
pT3b	25 (8.9%)	10 (9.6%)	15 (8.5%)	
pT4	0	0	0	
Pathological stage (N)				0.41
N0	77 (76.3%)	27 (75%)	50 (77%)	
N+	24 (23.7%)	9 (25%)	15 (23%)	
Number of lymph nodes removed at surgery	20.66 ± 7.91; 20 (2–47)	21.29 ± 11.24; 18.5 (2–47)	20.33 ± 5.52; 20 (12–47)	0.571
ISUP grading at surgery				0.262
1	36 (12.9%)	17 (16.4%)	19 (10.8%)	
2	138 (49.3%)	44 (42.3%)	94 (53.4%)	
3	67 (23.9%)	29 (27.9%)	38 (21.6%)	
4	18 (6.4%)	5 (4.8%)	13 (7.4%)	
5	21 (7.5%)	9 (8.7%)	12 (6.8%)	
Surgical margin at surgery (R)				<0.001
Negative	198 (71.5%)	59 (56.7%)	139 (80.3%)	
Positive	79 (28.6%)	45 (43.3%)	34 (19.7%)	
PNI at surgery				0.176
Negative	112 (40.1%)	36 (35%)	76 (43.2%)	
Positive	167 (59.9%)	67 (65%)	100 (56.8%)	
Cribriform/IDC at surgery				<0.001
Negative	236 (84%)	74 (71.8%)	162 (91.5%)	
Positive	44 (16%)	29 (28.2%)	15 (8.5%)	
Postoperative hospitalization (days)	4.15 ± 1.28; 4 (2–13)	3.93 ± 1.63; 4 (2–13)	4.3 ± 1; 4 (3–12)	0.025
Catheterization time (days)	10.23 ± 7.12; 10 (6–122)	10.35 ± 1.7; 10 (7–16)	10.16 ± 8.94; 10 (6–122)	0.831
Postoperative blood transfusion				0.6
No	277 (98.6%)	102 (98.1%)	175 (98.9%)	
Yes	4 (1.4%)	2 (1.9%)	2 (1.1%)	
Postoperative anastomotic stricture				0.6
No	277 (98.6%)	102 (98.1%)	175 (98.9%)	
Yes	4 (1.4%)	2 (1.9%)	2 (1.1%)	
Urinary incontinence				0.62
No	231 (82.2%)	87 (83.7%)	144 (81.4%)	
Yes	50 (17.8%)	17 (16.3%)	33 (18.6%)	
Erectile dysfunction				0.67
No	172 (61.2%)	62 (59.6%)	110 (62%)	
Yes	109 (38.8%)	42 (40.4%)	67 (38%)	
Biochemical progression				0.25
No	263 (90.2%)	100 (86.5%)	163 (92.1%)	
Yes	28 (9.8%)	14 (13.5%)	14 (7.9%)	
Time to biochemical progression (months)	6.65 ± 7.2; 4 (2–36)	5.91 ± 3.44; 5 (2–12)	7.33 ± 8.7; 4 (2–36)	0.48

Mean ± SD, median (range), number of cases (%).

**Table 2:** Risk for positive surgical margin, urethral stenosis, urinary incontinence, erectile dysfunction, and biochemical recurrence on the basis of bedside assistant expertise level.

Parameter	Univariate			Multivariate *		
	OR	95% CI	*p* Value	OR	95% CI	*p* Value
Positive surgical margin						
Attending group	Ref.			Ref.		
Resident group	0.32	0.18–0.55	<0.001	0.28	0.1–0-74	0.01
Urethral stenosis						
Attending group	Ref.			Ref.		
Resident group	0.3	0.27–3.33	0.3	0.36	0.03–4.34	0.4
Urinary incontinence						
Attending group	Ref.			Ref.		
Resident group	1.17	0.61–2.23	0.62	1.5	0.72–3.1	0.22
Erectile dysfunction						
Attending group	Ref.			Ref.		
Resident group	0.89	0.53–1.5	0.76	0.93	0.51–1.66	0.75
Biochemical recurrence						
Attending group	Ref.			Ref.		
Resident group	0.5	0.21–1.18	0.171	0.65	0.24–1.76	0.39

* Adjusted by preoperative PSA, risk class, biopsy multifocality, and cT stage.

**Table 3:** Differences in residents’ satisfaction in the two groups.

	Attending Group	Resident Group	*p* Value
Number	32	36	
Insight into surgical procedure	6.3 ± 2.8; 6 (3–9)	7.8 ± 2.2; 8 (5–10)	0.02
Confidence level	5.1 ± 2.4; 5 (1–8)	6.9 ± 1.6; 7 (4–9)	0.004
Gratification level	6.1 ± 1.8; 6 (3–8)	8.3 ± 1.6; 8 (6–10)	<0.001

All domains were evaluated with a score from 1 to 10. Values are expressed as mean ± SD and median (range).

## Data Availability

The raw data supporting the conclusions of this article will be made available by the authors on request.
